# Compositional tuning of phase interaction and strong magnetoelectric response in SRM–LNFO ceramic composites

**DOI:** 10.1039/d5ra07623d

**Published:** 2025-12-04

**Authors:** Pramod D. Mhase, Varsha C. Pujari, Sagar E. Shirsath, Sher Singh Meena, Abdul Ahad, Santosh S. Jadhav, Sunil M. Patange

**Affiliations:** a Department of Physics, Shri Krishna Mahavidyalaya Gunjoti Dharashiv Maharashtra 413 606 India smpatange@rediffmail.com; b School of Materials Science and Engineering, The University of New South Wales Sydney NSW 2052 Australia s.shirsath@unsw.edu.au shirsathsagar@hotmail.com; c Solid State Physics Division, Bhabha Atomic Research Centre Mumbai Maharashtra 400 085 India; d Department of Pharmaceutics, College of Pharmacy, King Saud University Riyadh 11451 Saudi Arabia; e D. S. M's Arts, Commerce & Science College Jintur Parbhani 431401 Maharashtra India

## Abstract

A novel series of magnetoelectric composites (1 − *x*)SrFe_12_O_19_–*x*La_0.5_Nd_0.5_FeO_3_ (*x* = 0.00–1.00) was synthesized *via* a citrate-assisted sol–gel method to explore the interplay between structural evolution and multifunctional properties. X-ray diffraction and Raman spectroscopy revealed phase coexistence with crystallite sizes increasing from 28 nm to 42 nm with rising perovskite content. Microstructural analysis showed particle sizes ranging from 0.19 µm to 0.54 µm, driven by compositional tuning of hexaferrite and perovskite phases. Mössbauer and XPS studies confirmed Fe^3+^ valence states and interfacial electronic interactions influencing magnetoelectric coupling. Magnetic characterization demonstrated a saturation magnetization decrease from 52.75 emu g^−1^ to 9.22 emu g^−1^ and a coercivity peak at 8.59 kOe for intermediate compositions due to exchange coupling effects. Ferroelectric measurements showed a remanent polarization of 1.21 µC cm^−2^ at *x* = 0.25 with corresponding leakage currents as low as 0.03 mA cm^−2^ for higher perovskite content. The optimized composition (*x* = 0.50) exhibited the highest magnetoelectric voltage output of 15.06 mV and coupling coefficient peak, highlighting efficient strain-mediated interphase interactions. These findings position SrFe_12_O_19_–La_0.5_Nd_0.5_FeO_3_ composites as promising candidates for magnetic sensors, multifunctional devices, and EMI shielding, providing valuable insights into structure–property relationships in oxide magnetoelectric composites.

## Abstract 2

A novel series of magnetoelectric composites (1 − *x*)SrFe_12_O_19_–*x*La_0.5_Nd_0.5_FeO_3_ (*x* = 0.00–1.00) was synthesized *via* a citrate-assisted sol–gel method to explore the interplay between structural evolution and multifunctional properties.

## Graphical abstract

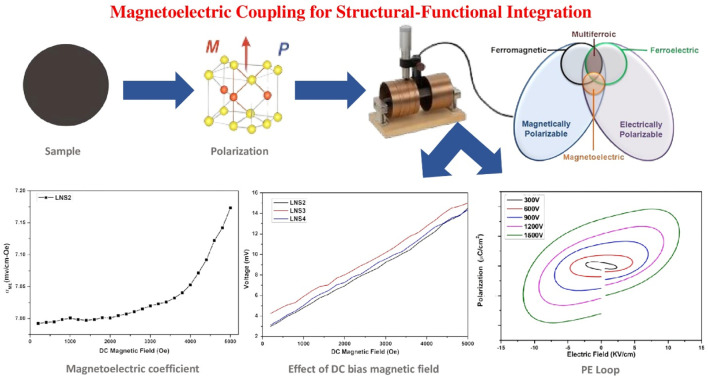

## 1. Introduction

The discovery of materials that combine magnetic, dielectric, and ferroelectric properties has attracted significant interest because of their promising applications in modern technologies, such as magnetic sensors, actuators, energy harvesters, and data storage devices.^1,2^ Magnetoelectric (ME) composites, which integrate magnetostrictive and piezoelectric phases, are especially appealing since they enable the control of magnetic states through electric fields and simultaneously influence electric polarization with magnetic fields.^3^ This dual manipulation opens up opportunities for creating compact, energy-efficient devices that overcome many challenges faced by single-phase multiferroics, including weak coupling at room temperature and complex fabrication processes.^4^ The search for multiferroic materials those exhibiting multiple ferroic orders simultaneously, continues to be a dynamic and exciting field in materials science and condensed matter physics. Materials with strong ME coupling not only broaden our understanding of fundamental mechanisms but also hold potential to revolutionize future technology.^5^ Hexaferrite–perovskite composites have garnered significant interest in recent years due to their unique combination of structural, magnetic, electric, and magnetoelectric properties. Strontium hexaferrite (SrFe_12_O_19_) is renowned for its high coercivity, moderate magnetization, and excellent chemical stability, making it a crucial material for applications such as permanent magnets, microwave absorption, and high-frequency devices. On the other hand, perovskite-type ferrites like La_0.5_Nd_0.5_FeO_3_ exhibit mixed ionic and electronic conductivity, strong dielectric properties, and the potential for magnetoelectric coupling, making them promising candidates for multifunctional material applications.^6,7^

Recent advancements in composite materials have demonstrated the effectiveness of combining hexaferrite and perovskite structures to enhance the overall physical properties of materials. Studies have highlighted the role of microstructural interactions, strain effects, and interfacial coupling in modulating the structural, dielectric, and magnetic responses of such composites.^8–10^ For instance, nanostructured perovskite-ferrite systems have been developed to optimize multiferroic behavior, enabling tailored electronic and magnetic functionalities.^11^ Magneto-polarization and magneto-dielectric studies on multiferroic (1 − *x*)Pb_0.75_Sm_0.25_TiO_3_–*x*Ba_0.7_Sr_0.3_Fe_12_O_19_ composites indicate extrinsic magnetoelectric coupling, primarily driven by Maxwell–Wagner interfacial polarization. Low Ba_0.7_Sr_0.3_Fe_12_O_19_ content showed the highest magneto-polarization (35.15% at 1.2 T), while equimolar composition (50–50) exhibited the strongest magneto-dielectric response (44.12% at 1.2 T, 100 Hz), highlighting the role of interfacial charge modulation under magnetic field.^12^ Dong Hun Kim *et al.* prepared a Composites of SrFe_12_O_19_ and perovskite La_0.7_Sr_0.3_MnO_3_, combining the hard magnetic properties and stability of SrFe_12_O_19_ with the colossal magnetoresistance of La_0.7_Sr_0.3_MnO_3_. SrFe_12_O_19_ and La_0.7_Sr_0.3_MnO_3_ exhibit *M*_s_ of 67.3 and 545 emu g^−1^, and *H*_c_ of 4776 and 78 Oe, respectively.^13^ Aditya Jain *et al.* reported that, introducing BiFeO_3_ at an optimal ratio has been shown to enhance the magnetoelectric coupling coefficient significantly, up to 11.9 mV (Oe^−1^ cm^−1^), which is approximately 1.5 times higher than that of pure BiFeO_3_, indicating the potential of phase substitution strategies for improving multiferroic behavior in BaFe_12_O_19_ (BaM)-based systems.^14^ Juan Liu *et al.* demonstrated the composite ceramics with significant improvements in ferroelectric performance, as evidenced by a decrease in the coercive field (*E*_c_ ≈ 30 kV cm^−1^) and an increase in remanent polarization (*P*_r_ ≈ 19.79 µC cm^−2^). The addition of BaM concurrently resulted in a linear increase in remanent magnetization, demonstrating how well BiFeO_3_–BaTiO_3_ phases work in conjunction with magnetic elements to maximize multiferroic behavior.^15^ Considering the growing demand for efficient magnetoelectric materials, the development of SrFe_12_O_19_(SRM)–La_0.5_Nd_0.5_FeO_3_(LNFO) composites represents a significant step forward in tailoring multifunctional properties through controlled synthesis and phase engineering. This approach is expected to yield novel material solutions for sensor devices, spintronic applications, and microwave absorption technologies.

Several recent studies have provided valuable insights into the development and characterization of hexaferrite and perovskite composites. A study by Shao *et al.* on hexaferrite composites revealed improved magnetic and dielectric properties, making them suitable for high-frequency applications. The electromagnetic wave absorber demonstrated outstanding absorption characteristics, achieving a reflection loss (RL) of −52 dB at a frequency of 29.5 GHz, with a corresponding thickness of 0.95 mm. The enhanced magnetocrystalline anisotropy and tunable permeability were found to significantly impact microwave absorption.^16^ Jacob Rosarian Joy S. *et al.* investigated magneto-dielectric and magneto-resistive behaviors in perovskite and hexaferrite composites, demonstrating strong coupling between dielectric and magnetic properties, suggesting their potential for multifunctional electronic devices.^17^ Recent research on magnetoelectric (ME) composites also shown that phase connectivity and interface engineering critically influence the strength of strain-mediated coupling between ferromagnetic and ferroelectric phases. A recent composite study of perovskite–ferrite nanocomposites such as Co_0.6_Cu_0.3_Zn_0.1_Fe_2_O_4_/Ba_0.9_Sr_0.1_Zr_0.1_Ti_0.9_O_3_,^18^ Ba_0.8_Sr_0.2_TiO_3_–Co_0.5_Cu_0.5_Fe_2_O_4_,^19^ Mn_0.5_Zn_0.5_Fe_2_O_4_–PbZr_*y*_Ti_1−*y*_O_3_,^20^ and Mn0_.5_Zn_0.5_Fe_2_O_4_–PbZr_0.5_Ti_0.5_O_3_ (ref. 21) achieved enhanced ME coupling by optimizing interface contact and phase dispersion; also demonstrated significant improvements in ME response through grain-boundary and interfacial strain control. Based on these observations, it is clear that careful tuning of phase proportions, microstructural connectivity, and interface coherence is essential for optimizing ME behavior with design strategy guiding the present investigation of SRM–LNFO composites.

This study presents a novel (1 − *x*)SrFe_12_O_19_–(*x*)La_0.5_Nd_0.5_FeO_3_ composite series synthesized by citrate-assisted sol–gel and optimized sintering. We investigate structural changes, phase coexistence, and how phase interactions influence magnetic, ferroelectric, and magnetoelectric properties, aiming to find the composition that maximizes ME coupling. Incorporating a rare-earth substituted perovskite into a hard hexaferrite matrix allows tuning of strain, lattice distortions, and charge dynamics for enhanced multifunctionality. Comprehensive characterization confirms well-formed composites with strong magnetoelectric response, improved magnetic anisotropy, and synergistic phase interactions. This work advances phase-engineered oxide composites for applications in magnetic sensors, spintronics, and EMI shielding.

## 2. Materials and methods

### 2.1. Materials

The chemical precursors employed in this study include strontium nitrate (Sr(NO_3_)_2_, 99%), ferric nitrate nonahydrate (Fe(NO_3_)_3_·9H_2_O, 98%), lanthanum nitrate hexahydrate (La(NO_3_)_3_·6H_2_O, 99%), citric acid (C_6_H_8_O_7_, 99%), neodymium nitrate hexahydrate (Nd(NO_3_)_3_·6H_2_O, 99.9%), and a 25% ammonia solution (NH_4_OH, extrapure). These reagents, all of analytical grade (AR), were procured from SRL, India. Their high purity ensures precise experimental conditions, reducing the risk of contamination and enhancing material consistency.

### 2.2. Preparation of materials

#### 2.2.1. Synthesis of SRM phase

The synthesis of SRM began by dissolving strontium nitrate (Sr(NO_3_)_2_) and ferric nitrate nonahydrate (Fe(NO_3_)_3_·9H_2_O) in 100 mL of deionized water. These precursors were combined in a stoichiometric ratio to maintain the intended chemical composition. Here, a stoichiometric amount of strontium nitrate (Sr(NO_3_)_2_: 4.7026 g), and iron(iii) nitrate nonahydrate (Fe(NO_3_)_3_·9H_2_O; 87.2375 g), were dissolved in 100 mL of deionized water. Citric acid (C_6_H_8_O_7_) was introduced in a 1 : 2 molar ratio relative to the metal nitrates to function as a chelating agent, ensuring homogeneous ion dispersion within the solution. The prepared solution was subjected to continuous stirring on a magnetic stirrer while being heated at 80 °C. Over a duration of 20–25 minutes, the uniformity of the precursor solution was achieved. To regulate the pH, a 25% NH_4_OH solution was gradually added dropwise until a pH of 7 was attained, which is optimal for gel formation. The temperature was then elevated to approximately 100 °C, facilitating the transition of the solution into a thick gel. This gel underwent auto-combustion, leading to the formation of fine powder through the decomposition of the metal–nitrate complex. The synthesized powder was thoroughly ground by hand with a mortar and pestle for 30 minutes to ensure a uniform particle size. It was then pre-sintered at 600 °C for 2 hours to remove residual moisture and impurities, making it suitable for subsequent processing.

#### 2.2.2. Synthesis of LNFO phase

To synthesize LNFO, a solution containing Lanthanum nitrate hexahydrate (La(NO_3_)_3_·6H_2_O), neodymium nitrate hexahydrate (Nd(NO_3_)_3_·6H_2_O), and ferric nitrate (Fe(NO_3_)_3_) was prepared by dissolving these precursors in 100 mL of deionized water while maintaining their stoichiometric proportions. Here, stoichiometric amounts of lanthanum nitrate hexahydrate (La(NO_3_)_3_·6H_2_O) 17.0115 g, neodymium nitrate nonahydrate (Nd(NO_3_)_3_·9H_2_O) 13.6915 g, and iron(iii) nitrate nonahydrate (Fe(NO_3_)_3_·9H_2_O) 48.36 g, were dissolved in 100 mL of deionized water. Citric acid (C_6_H_8_O_7_) was added in a 1 : 2 molar ratio concerning metal nitrates to stabilize the ions in solution. The resulting solution was continuously stirred on a magnetic stirrer at 80 °C for 20–25 minutes to ensure homogeneity. To maintain the desired pH of 7, a 25% NH_4_OH solution was introduced dropwise. The temperature was gradually increased to around 100 °C under continuous stirring, promoting the formation of a gel. This gel underwent self-combustion as the nitrates decomposed and volatile substances were released, resulting in a fine powder. The resulting powder was ground for 30 minutes to improve particle uniformity, followed by pre-sintering at 600 °C for 2 hours to eliminate moisture and residual impurities, yielding a purified material ready for further processing.

#### 2.2.3. Synthesis of magneto-electric composite (1 − *x*)SRM + (*x*)LNFO (*x* = 0.00, 0.25, 0.50, 0.75, 1.00)

A series of magneto-electric (ME) composites were prepared by blending nanocrystalline powders of (1 − *x*) SRM and (*x*) LNFO, with *x* values corresponding to 0.00 (LNS1), 0.25 (LNS2), 0.50 (LNS3), 0.75 (LNS4), and 1.00 (LNS5). Each powder underwent separate calcination before mixing to enhance crystallinity and eliminate unwanted phases. The powders were then thoroughly mixed using a mortar and pestle for 1 hour to achieve a homogeneously dispersed composite blend. To facilitate initial phase integration, the mixed powders were pre-sintered at 600 °C for 2 hours. Following this, the composite powders were further ground for an additional hour to ensure fine and uniform particle distribution. The final consolidation involved sintering at 1050 °C for 5 hours, with a controlled heating rate of 5 °C per minute. This meticulous thermal treatment promoted phase integration and densification, resulting in a well-formed magneto-electric composite suitable for subsequent characterization and analysis.

#### 2.2.4. Pellet fabrication for PE and ME measurements

To enable reliable ferroelectric, leakage and magnetoelectric (ME) testing, the as-synthesized powders were consolidated into disk-shaped pellets and processed as planar capacitor devices. Approximately 2 g of composite powder was homogeneously mixed with 2–3 wt% aqueous PVA solution and thoroughly ground to ensure uniform distribution. The mixture was then pressed into circular pellets of 10 mm diameter using a hydraulic press at 5 tons. The pellets were air-dried and sintered at 500 °C for 2 h to remove the PVA binder completely and to improve mechanical integrity. Both flat faces of each pellet were coated with conductive silver paste and cured at 150 °C for 30 min to form uniform electrodes. These silver-coated pellets served as planar capacitor devices for electrical and ME testing.

#### 2.2.5. Characterization techniques

The structural properties of the ME composites were investigated using X-ray diffraction (XRD) with Cu-K_α_ radiation (*λ* = 1.5406 × 10^−10^ m) over a 2*θ* range of 20°–80°. The collected diffraction patterns were analyzed and refined using FullProf software to determine phase composition and crystallographic parameters. The surface morphology and grain structure were studied using FESEM (Hitachi S-4800) in backscattered electron imaging mode. Grain sizes were determined using the linear intercept method in ImageJ software. Raman spectroscopic analysis was performed using a RENISHAW spectrometer in the range of 100–800 cm^−1^ to evaluate the vibrational modes and phase identification of the materials. Mössbauer spectroscopy (MS) was conducted at room temperature in transmission geometry using a Co^57^ source embedded in a Rh matrix (5 mCi). The velocity scale was calibrated with an α-^57^Fe metal foil reference. The recorded spectra were analyzed using the WinNormos software to determine hyperfine parameters, including isomer shift, quadrupole splitting, and magnetic hyperfine field distribution. Magnetic characteristics were analyzed using a Vibrating Sample Magnetometer (VSM) (Microsense, Model ADE – EV9) to measure hysteresis loops and determine parameters such as saturation magnetization and coercivity. The *P*–*E* hysteresis loop measurements, current–voltage characteristics, and magnetoelectric coupling measurements for the composite samples were performed using the magnetoelectric coupling measurement system developed by Marine India Technologies.

## 3. Results and discussion

### 3.1. Structural analysis

**Fig. 1:**
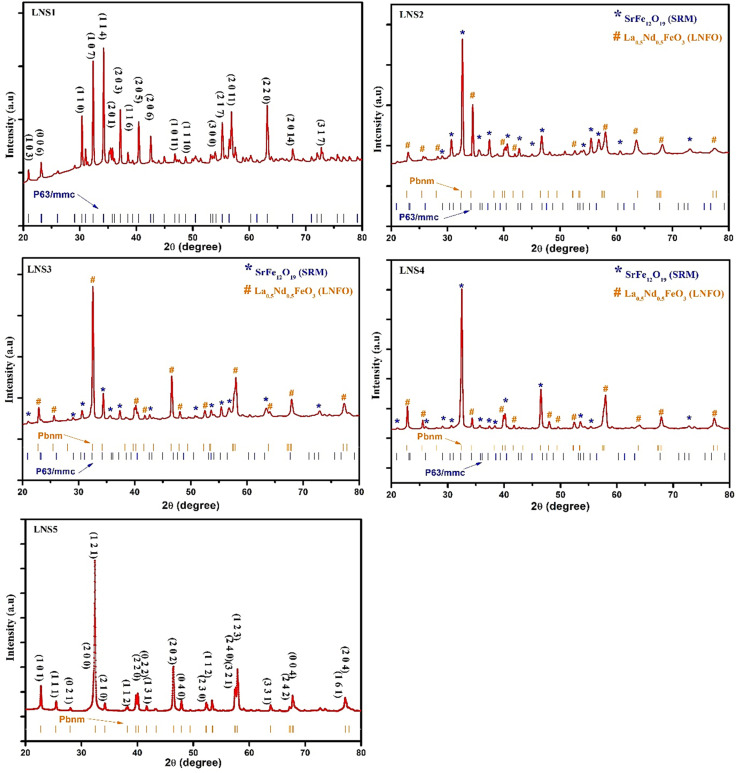
XRD plot of (1 − *x*)SRM–(*x*)LNFO (LNS1 to LNS5) samples.

The XRD patterns (Fig. 1) of (1 − *x*)SRM–(*x*)LNFO composites (LNS1–LNS5) recorded over a 2*θ* range of 20°–80° confirmed the presence of both M-type hexaferrite (SRM, space group *P*6_3_/*mmc*) and orthorhombic perovskite (LNFO, space group *Pbnm*) phases. The relative intensities of the diffraction peaks varied with composition, indicating a progressive phase evolution from ferrite-rich (LNS1) to perovskite-rich (LNS5) systems. For LNS1 (*x* = 0.00), lattice constants were found to be *a* = *b* = 5.88 Å and *c* = 23.05 Å, while increasing LNFO content introduced minor shifts in these parameters due to lattice strain and phase interaction.^22^ The perovskite phase exhibited stable lattice constants of *a* ≈ 5.56 Å, *b* ≈ 7.81 Å, and *c* ≈ 5.50 Å.

Crystallite size (*D*) was calculated using the Scherrer equation:^23^1
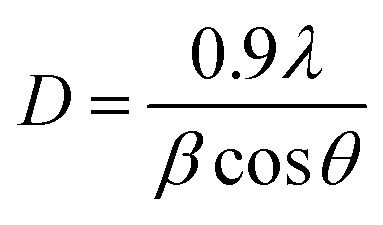


For LNS1, the average crystallite size was estimated to be ∼28 nm. As the LNFO content increased, the crystallite size grew gradually, reaching ∼42 nm for LNS5, suggesting enhanced crystallization and reduced structural disorder with rising perovskite content. The gradual evolution in lattice parameters and crystallite size indicates a well-defined structural transition within the composite series.^24^ Such tunable structural characteristics are expected to play a critical role in tailoring the ferroelectric and magnetoelectric properties of the system.^1^ Furthermore, the clear presence of both magnetic (SRM) and dielectric (LNFO) phases validates the composite architecture, crucial for achieving multifunctional behavior.

### 3.2. Microstructural analysis

**Fig. 2:**
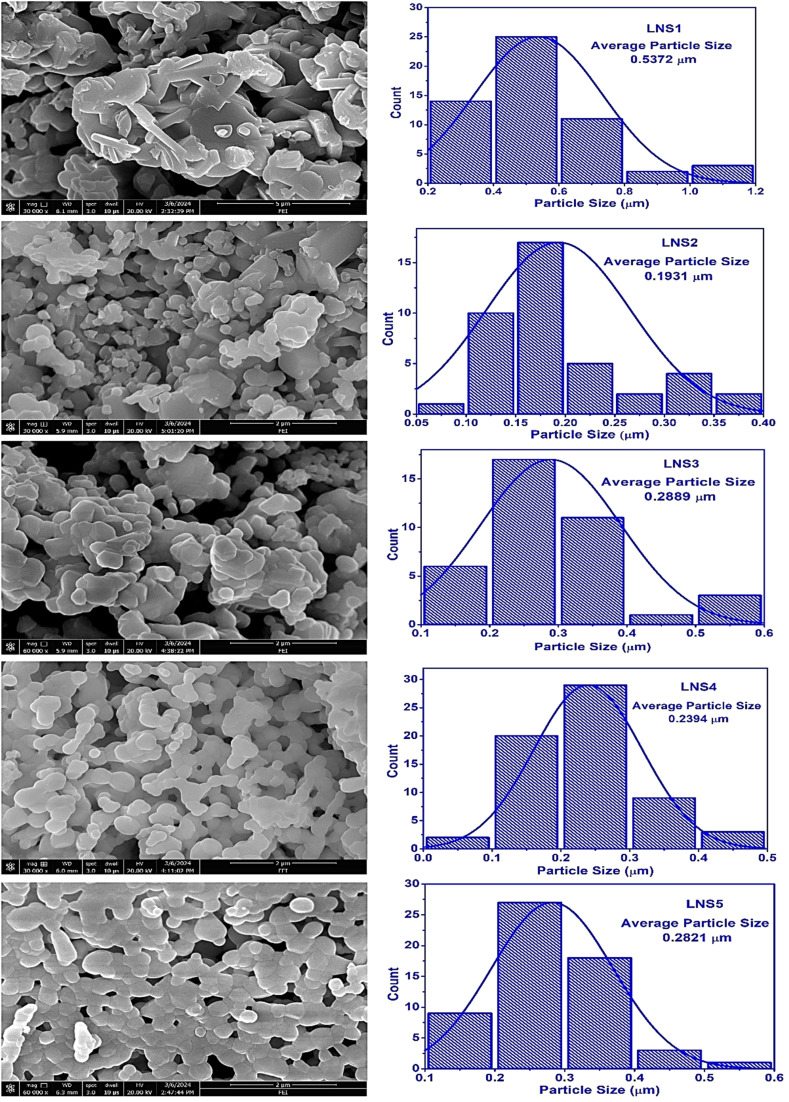
FESEM micrographs (1 − *x*)SRM–(*x*)LNFO (LNS1 to LNS5) composites.

The FESEM micrographs of (1 − *x*)SRM–(*x*)LNFO (LNS1 to LNS5) composites reveal a composition-dependent evolution in morphology, particle size, and grain connectivity. The particle size was quantitatively analyzed using ImageJ software. The calculated particle size distribution for each composition is presented in the form of histograms (Fig. 2). The variation in microstructure with increasing LNFO content can be attributed to the distinct crystallization behavior of the hexaferrite and perovskite phases, which influences grain growth kinetics, interfacial interactions, and densification mechanisms. The average particle sizes obtained from the histogram analysis varies between 0.1931 µm to 0.5372 µm. The observed variation in particle size is attributed to the influence of phase composition, sintering behavior, and diffusion dynamics within the composite system.^25^

The sharp decrease in particle size from LNS1 (0.5372 µm) to LNS2 (0.1931 µm) is due to the introduction of the perovskite LNFO phase, which disrupts the natural platelet-like growth of hexaferrite grains.^26^ The limited diffusion of Fe^3+^ and Sr^2+^ ions in the presence of La^3+^ and Nd^3+^ results in a restriction of grain boundary movement, leading to a finer microstructure. The slight increase in particle size for LNS3 (0.2889 µm) suggests a competitive effect between grain refinement and coarsening mechanisms.^27^ The coexistence of both hexaferrite and perovskite phases at this composition leads to a localized enhancement of Fe ion diffusion, allowing moderate grain growth.^28^ The minor variation in particle size for LNS4 (0.2394 µm) and LNS5 (0.2821 µm) indicates a gradual transition to a perovskite-dominated microstructure. The increase in La^3+^ and Nd^3+^ ion concentration enhances sintering behavior, promoting higher densification and controlled grain growth.^28^ This systematic microstructural evolution, as validated by ImageJ software analysis and histogram representation, demonstrates the tunability of grain morphology through compositional engineering, which is crucial for optimizing the multifunctional properties of ceramic composites. The phase composition and distribution were further assessed based on the XRD and FESEM results. The relative diffraction intensities confirm the coexistence of SRM and LNFO phases in proportions close to their nominal stoichiometry. The FESEM micrographs reveal a uniform dispersion of fine LNFO grains embedded within the ferrite matrix, suggesting a 0–3 connectivity, where discrete ferroelectric regions are interpenetrated by a continuous magnetostrictive phase.^29^ Such a configuration facilitates effective strain transfer across interfaces, thereby promoting strong magnetoelectric coupling.

### 3.3. Raman spectroscopy

**Fig. 3:**
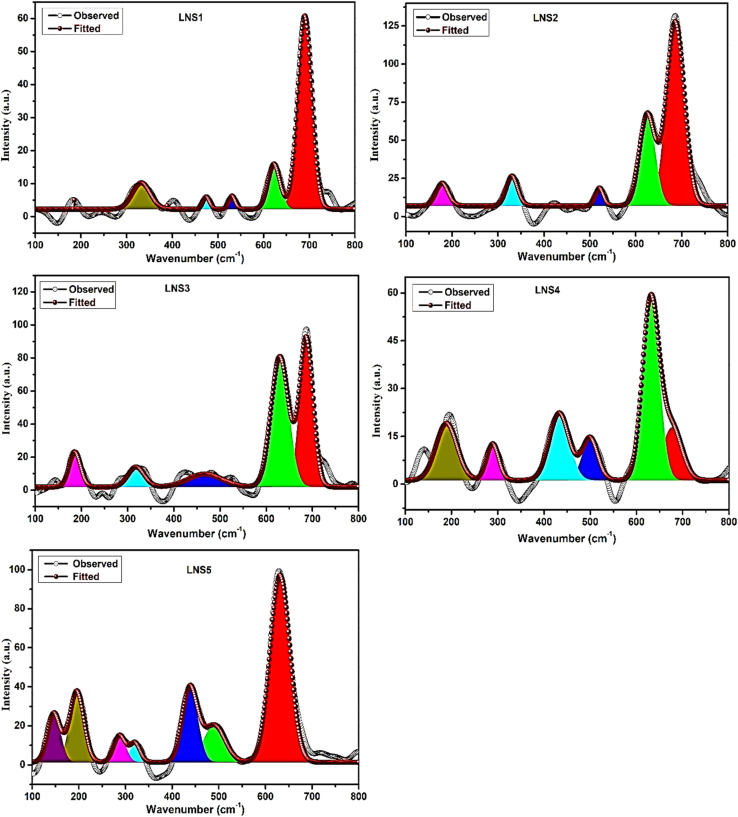
Deconvolution of Raman spectra with Gaussian fitting of (1 − *x*)SRM–(*x*)LNFO composites.

The Raman spectra of (1 − *x*)SRM–(*x*)LNFO composites (*x* = 0.00, 0.25, 0.50, 0.75, 1.00) were recorded to investigate vibrational modes and structural modifications due to compositional variation. The Raman shifts observed in the spectra are as depicted in Fig. 3. In the case of pure SRM (*x* = 0.00), the characteristic vibrational modes of hexaferrite were observed, with strong peaks appearing at 621.32 cm^−1^, 689.77 cm^−1^ wavenumbers corresponding to Fe–O stretching and bending vibrations within the Fe^(4)^O_6_ octahedral and Fe^(3)^O_4_ tetrahedral units respectively.^30^ For pure LNFO (*x* = 1.00), the Raman modes were dominated by perovskite-type phonon vibrations associated with Fe–O bond stretching and lattice vibrations.^31^ With increasing *x*, a systematic shift in Raman peaks was observed, indicating structural distortions due to the substitution of LNFO into the SRM matrix.

The Raman peak around 332 cm^−1^ associated with tetrahedral Fe vibrations shifted down around 186–195 cm^−1^ in the composites, indicating structural strain effects.^32^ The disappearance of the mode around 404 cm^−1^ in some compositions suggests phonon mode damping due to increased disorder.^33^ The peak around 530 cm^−1^, assigned to bending vibrations of Fe–O bonds, shifted slightly supporting local distortions in the lattice.^34^ The mode around 689 cm^−1^, a characteristic feature of hexaferrite, showed a progressive decrease approximately around 2–6 cm^−1^ shift, revealing an interaction between the perovskite and hexaferrite phases. The broadening of peaks with increasing *x*, suggesting increased structural disorder and phonon confinement effects due to phase mixing.^35^

The coexistence of Raman modes from both hexaferrite and perovskite structures in intermediate compositions (*x* = 0.25, 0.50, 0.75) confirms the formation of composite phases with significant lattice strain. Overall, the Raman analysis confirms the structural evolution and phase interaction between SRM and LNFO in the composite system, supporting the presence of vibrational signatures from both parent phases. These findings align well with XRD results and provide further insight into the bonding characteristics and lattice distortions occurring with increasing LNFO content.

### 3.4. X-ray photoelectron spectroscopy (XPS) analysis

**Fig. 4:**
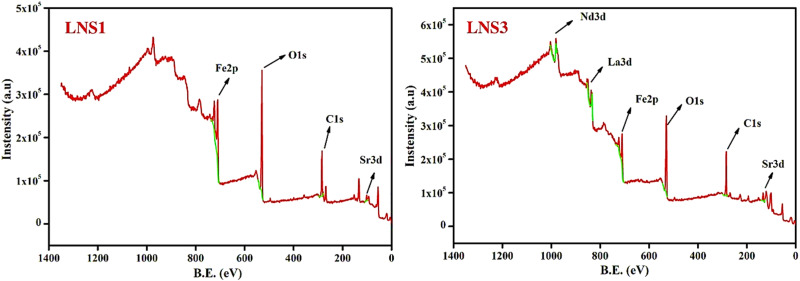
XPS survey spectra for LNS1 and LNS3.

**Fig. 5:**
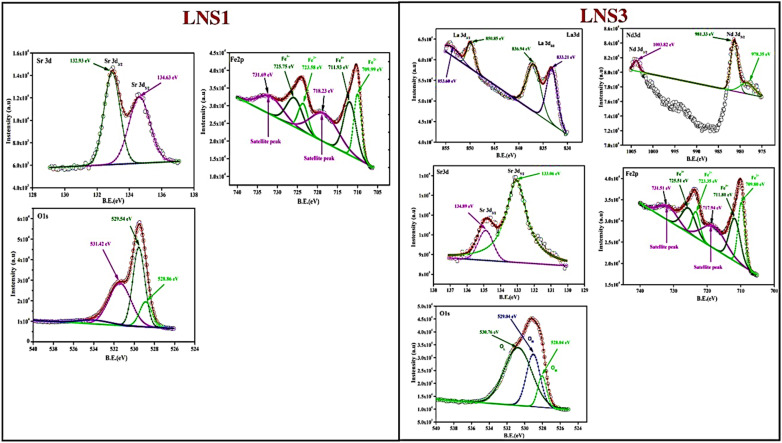
Deconvoluted XPS spectra for LNS1 and LNS3.

The surface chemical composition and oxidation states of elements in (1 − *x*)SRM–(*x*)LNFO composites were investigated using XPS. Deconvoluted XPS spectra provided insight into the bonding characteristics and electronic states of Fe, Sr, La, Nd, and O elements within the composite system. Fig. 4 presents the XPS survey spectra of LNS1 and LNS3, confirming the presence of Fe, Sr, O, La, and Nd elements, ensuring the phase purity of the synthesized composites. The survey spectra indicate strong Fe 2p, Sr 3d, and O 1s peaks in LNS1. In contrast, additional La 3d and Nd 3d peaks are evident in LNS3, confirming successful incorporation of the perovskite phase into the hexaferrite matrix. To further analyze the electronic states of these elements, the high-resolution XPS spectra for Fe 2p, Sr 3d, La 3d, Nd 3d, and O 1s were deconvoluted, as shown in Fig. 5.

The Fe 2p spectra for LNS1 (pure SRM) and LNS3 ((0.5)SRM–(0.5)LNFO) exhibited characteristic peaks corresponding to Fe^3+^ oxidation states. For LNS1, the Fe 2p_3/2_ peak was observed at 711.93 eV, while the Fe 2p_1/2_ peak appeared at 725.75 eV, confirming the presence of Fe^3+^ as the dominant oxidation state.^36^ Additionally, satellite peaks at 718.23 eV and 731.69 eV further substantiated the Fe^3+^ state, aligning well with literature-reported values.^37^

The incorporation of LNFO in LNS3, a slight shift in peak positions was observed, with the Fe 2p_3/2_ peak appearing at 711.80 eV and the Fe 2p_1/2_ peak at 725.51 eV. This shift suggests electronic interactions between hexaferrite and perovskite phases, influencing the Fe oxidation environment. The presence of mixed Fe^3+^ states suggests that charge compensation mechanisms occur at the interfacial regions of the composite system.^38^ The slight shift in satellite peak position (from 718.23 eV in LNS1 to 717.94 eV in LNS3) suggests changes in charge transfer effects between Fe 3d and O 2p orbitals due to the incorporation of LNFO. The decrease in satellite peak intensity in LNS3 may indicate an increase in electronic interactions at the hexaferrite–perovskite interface, affecting charge localization and orbital hybridization.

The Sr 3d spectra of LNS1 exhibited two peaks at 134.63 eV (Sr 3d_3/2_) and 132.93 eV (Sr 3d_5/2_), confirming the Sr^2+^ oxidation state characteristic of SRM.^39^ In LNS3, the Sr 3d binding energy showed a minor shift to 134.88 eV and 133.06 eV, which may be attributed to slight structural distortions introduced by La and Nd substitution.^40^ In LNS3, the La 3d_5/2_ and La 3d_3/2_ peaks were observed at 833.21 eV and 849.85 eV, respectively, confirming the La^3+^ oxidation state. Similarly, the Nd 3d_5/2_ peak at 978.35 eV and Nd 3d_3/2_ peak at 1003.82 eV indicated that Nd also exists in a stable trivalent state. These findings are consistent with previous reports on rare-earth-doped perovskite systems.^41^ The presence of La^3+^ and Nd^3+^ suggests that their incorporation into the composite structure occurs without significant alteration of their oxidation states. The O 1s spectra provided valuable insight into the oxygen bonding environment in the composites. For LNS1, the O 1s peak at 529.54 eV corresponded to lattice oxygen (O^2−^), while additional peaks at 531.42 eV indicated surface-adsorbed oxygen species.^42^ In LNS3, the peak at 530.76 eV suggested the presence of oxygen vacancies and hydroxyl groups, which can contribute to enhanced dielectric and magnetoelectric properties in the composite system. The binding energy shifts in O 1s peaks highlight the influence of LNFO incorporation on oxygen bonding and defect states. The slight shifts in binding energies values suggest electronic interactions between hexaferrite and perovskite phases, which may influence the material's magnetic and dielectric behavior.

The structural results reveal a clear correlation between composition, microstructure, and multifunctional performance in the (1 − *x*)SRM–(*x*)LNFO composites. Increasing LNFO content refines grain size and enhances interfacial connectivity, promoting efficient strain and charge transfer across ferrite–perovskite boundaries. The coexistence of Fe^2+^/Fe^3+^ states and lattice distortion confirmed by XPS facilitate charge compensation and local polarization. Consequently, intermediate compositions (LNS2–LNS4) exhibit optimized structure–property coupling, underscoring the intrinsic composition–microstructure–performance interrelationship. The EDS and XPS analyses corroborate the uniform distribution of constituent elements and the stable coexistence of Fe^2+^/Fe^3+^ oxidation states, which play a crucial role in facilitating charge compensation and local polarization. Thus, the collective interpretation of the structural and compositional data indicates that the balanced phase ratio in the intermediate compositions provides the most favorable conditions for efficient coupling. The optimized interface connectivity, controlled lattice distortion, and stable mixed-valence Fe states together enable effective strain and charge transfer across the ferrite–perovskite boundary. This intrinsic correlation between composition, microstructure, and electronic structure directly governs the enhanced ferroelectric and magnetoelectric responses as discussed in the subsequent sections.

### 3.5. Mössbauer spectroscopy

**Fig. 6:**
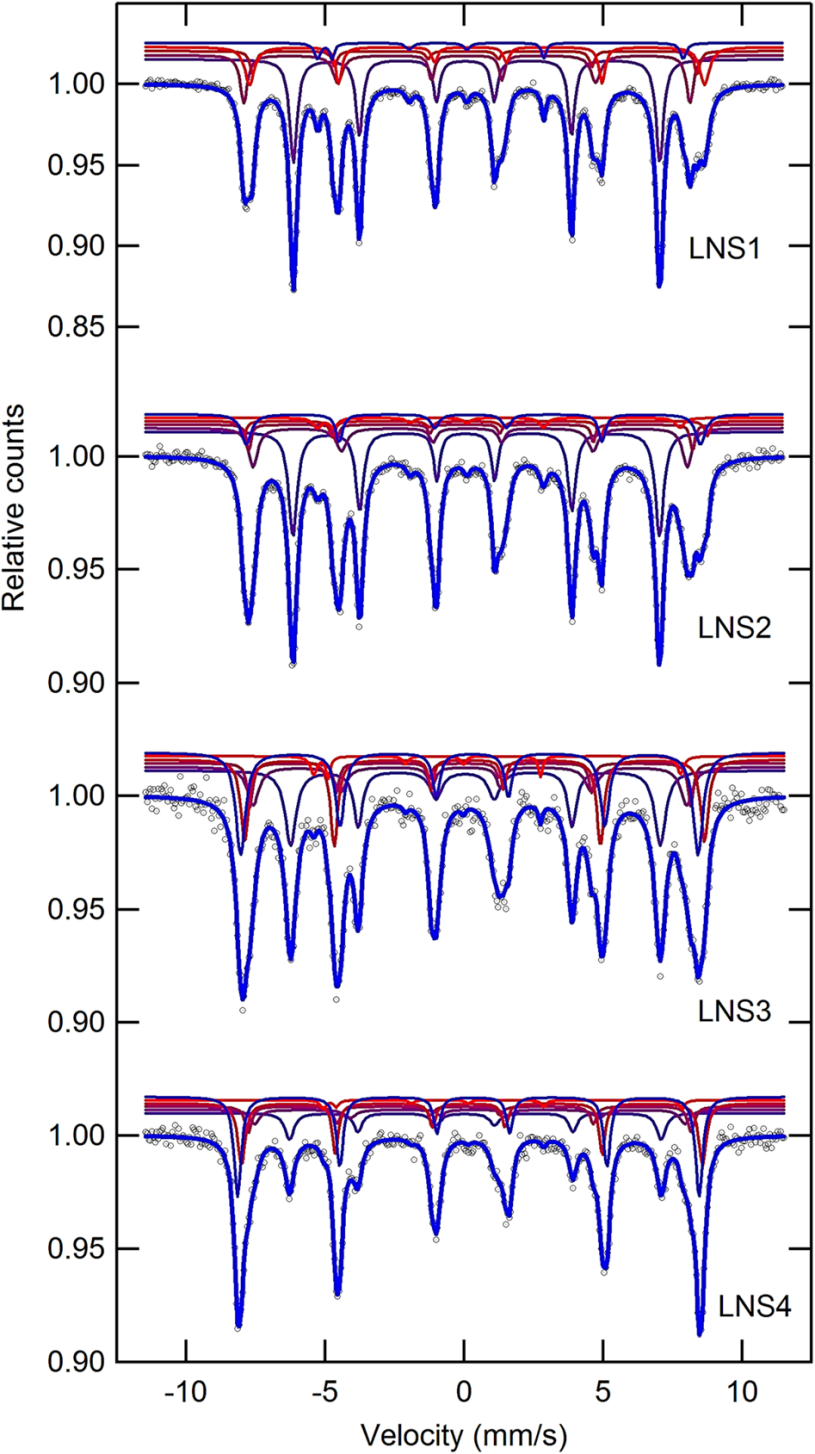
Room temperature Mössbauer spectra of (1 − *x*)SRM–(*x*)LNFO composite samples for LNS1, LNS2, LNS3, and LNS4.

Mössbauer spectroscopy was employed to investigate the local magnetic environment of Fe ions in the (1 − *x*)SRM–(*x*)LNFO composite system, where *x* = 0.00 (LNS1) to 0.75 (LNS4) at ambient temperature, as shown in Fig. 6. The spectra were analysed using magnetic sextets corresponding to Fe sites in SRM, and a paramagnetic phase associated with LNFO. The evolution of the hyperfine parameters Relative Area (RA), Outer Line Width (OLW), Isomer Shift (IS), Quadrupole Splitting (QS), and Hyperfine Magnetic Field (*B*_hf_) provides insight into the phase distribution and local environments of Fe ions as shown in Table 1. In LNS1 (*x* = 0.00), the spectrum is fully described by five sextets attributed to Fe^3+^ at crystallographic sites in M-type SRM with 12k, 4f_1_, 2a, 4f_2_, and 2b.^43^ As *x* increases, a sixth spectral component appears, identified as a sextet from the LNFO phase. The RA of this sextet grows from 20.85% in LNS2 to 36.97% in LNS4, confirming complete transformation to the perovskite phase. The decrease in RA of the SRM sextets correlates with progressive dilution of the magnetic phase. The outer line widths for SRM components remain narrow in LNS1, reflecting minimal local disorder. Among these, the 2b↑ site consistently exhibits broader line widths of approximately 0.270–0.286 mm s^−1^, indicative of a more distorted environment. As LNFO content increases, the sextet from the LNFO phase also shows modest broadening as 0.315 mm s^−1^ in LNS4, suggesting increased strain or interface effects in the composite structure.

**Table 1:** Hyperfine parameters Relative Area (*R*_A_), Outer Line Width (OLW), Isomer Shift (IS), Quadrupole Splitting (QS), and Hyperfine Magnetic Field (*B*_hf_), Goodness of fit (*χ*^2^)

Sample composition	Fe-sites and spin orientation	Relative area (*R*_A_) %	Outer line width (mm s^−1^) (±0.005)	Isomer shift (mm s^−1^) (±0.005)	Quadrupole splitting (mm s^−1^) (±0.005)	Hyperfine magnetic field (Tesla) (±0.02)	Goodness of fit (*χ*^2^)
LNS1	12k↑	49.27	0.324	0.364	0.404	40.89	2.39
	4f_1_↓	18.95	0.309	0.211	0.009	49.78	
	2a↑	06.14	0.230	0.294	0.421	49.61	
	4f_2_↓	18.95	0.419	0.464	0.240	50.79	
	2b↑	06.69	0.270	0.297	2.245	40.81	
LNS 2	12k↑	44.96	0.370	0.364	0.377	40.92	1.57
	4f_1_↓	16.32	0.384	0.282	0.097	48.39	
	2a↑	05.57	0.230	0.242	0.194	49.49	
	4f_2_↓	06.58	0.209	0.361	0.321	51.77	
	2b↑	05.72	0.360	0.278	2.162	40.43	
	LNFO	20.85	0.457	0.401	0.100	50.66	
LNS 3	12k↑	37.86	0.475	0.337	0.370	41.25	0.88
	4f_1_↓	12.68	0.507	0.251	0.140	48.40	
	2a↑	04.71	0.230	0.225	0.229	49.21	
	4f_2_↓	13.55	0.338	0.361	0.262	51.33	
	2b↑	02.52	0.286	0.173	2.256	40.94	
	LNFO	28.68	0.429	0.358	−0.108	51.06	
LNS 4	12k↑	20.49	0.397	0.340	0.358	41.48	0.90
	4f_1_↓	08.25	0.416	0.409	−0.185	48.00	
	2a↑	09.04	0.288	0.237	0.164	49.41	
	4f_2_↓	23.1	0.322	0.342	0.116	51.46	
	2b↑	02.15	0.230	0.375	2.253	40.05	
	LNFO	36.97	0.315	0.358	−0.179	51.51	
LNS5	LNFO	100.00	0.312	0.356	−0.048	51.74	1.08

The isomer shift (IS) values across all samples range from 0.173 to 0.464 mm s^−1^, confirming the presence of high-spin Fe^3+^ in both SRM and LNFO phases. The IS for Fe sites in SRM shows slight variations with increasing LNFO content, indicating minor changes in electron density due to structural distortion or interface strain. The LNFO component, represented by a sextet from the LNFO phase, shows relatively stable IS values between 0.356 to 0.401 mm s^−1^, consistent with Fe^3+^ in an octahedral environment. These trends reflect subtle electronic rearrangements during phase evolution in the composite system.^44^

The quadrupole splitting (QS) values in the (1 − *x*)SRM–(*x*)LNFO composite system exhibit a wide range from −0.179 mm s^−1^ to 2.445 mm s^−1^, indicating varying degrees of local symmetry around the Fe nuclei. In SRM – rich samples LNS1–LNS4, large QS values, particularly for the 2b site at 2.162 to 2.256 mm s^−1^ in for LNS1 with composites (LNS2–LNS4) and reflect significant local distortion or asymmetry, likely due to Jahn–Teller effects or octahedral tilting.^45^ In contrast, the LNFO component (LNS4) shows much smaller QS values, approaching −0.179 mm s^−1^, indicating a more symmetrical environment around Fe in the perovskite phase. These variations suggest phase-specific electronic and geometric distortions within the composite, with the hexaferrite phase experiencing stronger local asymmetry than the perovskite phase.^46^ The hyperfine magnetic field (*B*_hf_) values in the (1 − *x*)SRM–(*x*)LNFO composite system range from 40.05 to 51.77 T, indicating strong magnetic ordering in the SRM phase. The highest *B*_hf_ values up to ∼51.7 T are observed for Fe^3+^ at octahedral sites such as 4f_2_ and 2a, consistent with the ferrimagnetic nature of M-type hexaferrite. As the LNFO content increases, the intensity of magnetic sextets decreases, but *B*_hf_ values remain high for the remaining SRM components, suggesting that magnetic dilution occurs without significant loss of internal field strength.^1^ In the fully perovskite sample LNS5, the absence of magnetic splitting confirms the paramagnetic nature of LNFO at room temperature, as reported previously.^47^ These results support a gradual ferrimagnetic-to-paramagnetic transition with increasing *x*, while preserving strong local magnetic fields in the hexaferrite phase.^48^

These findings confirm that Mössbauer spectroscopy is a sensitive probe of the composite's phase evolution and it underscoring how the long-range magnetic order of SRM is gradually disrupted by the increasing incorporation of the LNFO phase. Such tunability of the magnetic and electronic environment is critical for multifunctional applications, including magneto-electronics, spin filters, and sensor materials.

### 3.6. VSM analysis

**Fig. 7:**
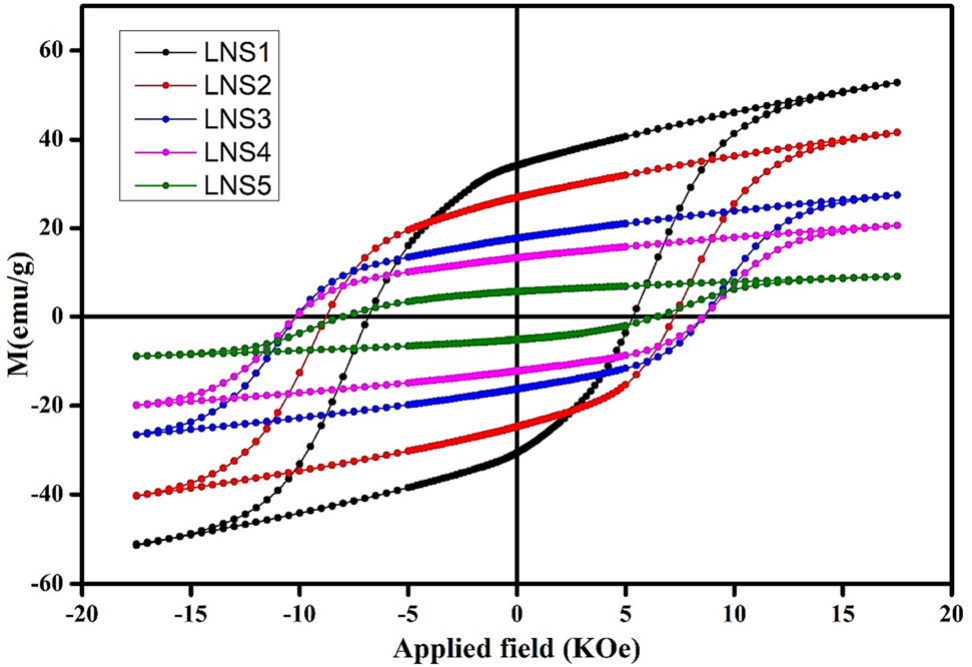
Hysteresis loops for all the prepared composites of (1 − *x*)SRM–(*x*)LNFO.

The magnetic properties of the (1 − *x*)SRM–(*x*)LNFO composite system (where *x* = 0.00 to 1.00) were investigated using VSM at room temperature. Fig. 7 demonstrates a hysteresis loop for VSM analysis, whereas hysteresis parameters including saturation magnetization (*M*_s_), remanent magnetization (*M*_r_), coercivity (*H*_c_), and squareness ratio (*M*_r_/*M*_s_) are summarized in Table 2. A systematic variation in magnetic properties with increasing concentration of LNFO phase is observed. The saturation magnetization (*M*_s_) decreases progressively from 52.75 emu g^−1^ for LNS1 (Pure SRM) to 9.22 emu g^−1^ for LNS5 (Pure perovskite LNFO). This decline can be attributed to the gradual dilution of the hard magnetic SRM phase with the weakly magnetic orthoferrite phase, LNFO, which inherently possesses a lower magnetic moment due to its canted antiferromagnetic ordering.^49^ A similar trend is noted in the remanent magnetization (*M*_r_), which decreases from 34.14 emu g^−1^ in LNS1 to 5.71 emu g^−1^ in LNFO. This trend aligns with the Mössbauer spectroscopy results, where the relative area of the LNFO sextet increased systematically. The reduction in both *M*_s_ and *M*_r_ confirms the dominance of the weakly magnetic LNFO phase as its proportion increases in the composite, thereby weakening the net magnetization.^50^

**Table 2:** Magnetic parameters obtained from VSM at room temperature

Sample code	Coercivity (*H*_c_) (KOe)	Magnetization (*M*_s_) (emu g^−1^)	Retentivity (*M*_r_) (emu g^−1^)	Squareness ratio = (*M*_r_/*M*_s_)
LNS1	5.339	52.75	34.14	0.647
LNS2	7.247	41.53	26.95	0.649
LNS3	8.585	27.39	17.81	0.650
LNS4	8.59	20.57	13.22	0.643
LNS5	6.487	9.22	5.71	0.619

Interestingly, the coercivity (*H*_c_) displays a non-linear behavior. It initially increases from 5.339 kOe in LNS1 to a peak of 8.590 KOe in LNS4, before slightly decreasing to 6.487 kOe in LNS5. The initial rise in *H*_c_ can be associated with the exchange coupling between the hard SRM and soft LNFO phases, which can enhance magnetic anisotropy.^51^ It is also evidenced by the broadening of Mössbauer sextets in which the outer line width (OLW) of the sextets broadens for intermediate compositions, reflecting local lattice distortions. However, beyond a certain threshold (LNS4), the dominance of the soft phase may weaken the interfacial exchange interactions, resulting in reduced coercivity.

The squareness ratio (*M*_r_/*M*_s_) remains nearly constant for LNS1 to LNS4, indicating stable uniaxial anisotropy typical of SRM dominated composites. The slight decrease for LNS5 further corroborates the transition to a weakly magnetic perovskite phase. The consistency of (*M*_r_/*M*_s_) in LNS1–LNS4 implies minimal interphase exchange coupling, as expected for a composite with distinct magnetic and paramagnetic phases. The VSM data complement the Mössbauer hyperfine analysis, where the reduction in *M*_s_ correlates with the declining relative area of SRM sextets. The anomalous *H*_c_ peak in LNS4 aligns with the broadening of Mössbauer OLW, suggesting strain-induced anisotropy at intermediate compositions.^52^ These results collectively highlight the interplay between phase fraction, interfacial effects, and magnetic properties in the composite system.

### 3.7. Ferroelectric properties

#### 3.7.1. Polarization–electric field (*P*–*E* loop) characteristics

**Fig. 8:**
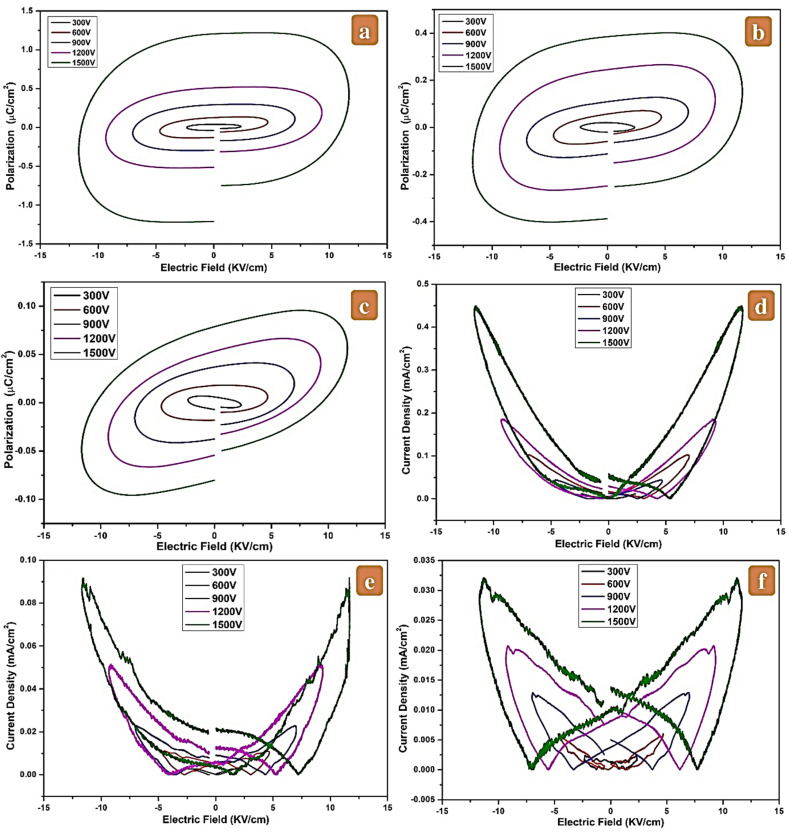
(a) *P*–*E* loop of LNS2, (b) *P*–*E* loop of LNS3, (c) *P*–*E* loop of LNS4, (d) *J*–*E* curve for LNS2, (e) *J*–*E* curve for LNS3, and (f) *J*–*E* curve for LNS4.

The ferroelectric characteristics of the synthesized (1 − *x*)SRM–(*x*)LNFO composites with varying compositions (*x* = 0.25, 0.50, and 0.75) were investigated by measuring the polarization–electric field (*P*–*E*) hysteresis loops at room temperature. The measurements were performed using silver-paste-electroded pellets at an applied electric field of ±1.5 kV cm^−1^ and a frequency of 1 kHz. All composite samples exhibit ferroelectric behavior, as shown in Fig. 8(a)–(c); however, the loops are still unsaturated. The influence of leakage current is responsible for the slight opening observed in each loop.^53^

Fig. 8(a) illustrates the *P*–*E* loops obtained for the LNS2 sample, which exhibits a elliptical and symmetric hysteresis loop indicative of lossy ferroelectric behavior. The LNS2 composition, with a higher content of the hard magnetic SRM phase, demonstrated the highest remanent polarization (P_r_) of 1.21 µC cm^−2^, a coercive field (*E*_c_) of 11.70 kV cm^−1^, and a maximum polarization (*P*_s_) of 1.23 µC cm^−2^ under the applied field. This suggests the presence of a polarizable component within the microstructure, likely arising from interfacial polarization between the ferrite and perovskite phases.^54^

As the content of LNFO increases (as in LNS3 and LNS4), a noticeable reduction in ferroelectric parameters (*P*_r_, *P*_s_, and *E*_c_) was observed, as shown in Fig. 8(b) and (c). This trend indicates a dilution of ferroelectric switching behavior, possibly due to the increased contribution of the perovskite phase, which exhibits weaker long-range ferroelectric order under the given field conditions.^55^ Furthermore, the decrease in polarization values with increasing perovskite phase (*x*) may also be attributed to increased leakage currents or reduced domain alignment as the magnetic phase fraction decreases.^56^ Also, the electric polarization of the composites decreases with increasing LNFO component because the addition of perovskite material alters the domain wall mobility coupling and domain position while suppressing the electric polarization vectors.^57,58^ As mentioned earlier, although the non-reinforced sample exhibited higher ferroelectric characteristics, both the FE and FM phases are necessary to achieve magnetodielectric features.^59^

As a result, the observed ferroelectric behavior confirms the coexistence of polar and magnetic phases, validating the multiferroic nature of the composites. However, the weak loop saturation and elliptical shape suggest lossy and leaky dielectric behavior, which may limit the utility of these materials in practical ferroelectric memory applications but still holds potential for magnetoelectric and multifunctional devices.

#### 3.7.2. Current density–electric field (*J*–*E*) characteristics

The leakage current behavior of the (1 − *x*)SRM–(*x*)LNFO composites was investigated by measuring the current density (*J*) as a function of the applied electric field (*E*) under a bipolar sweeping voltage in the range of ±15 kV cm^−1^, with corresponding voltages of 300 V to 1500 V at a frequency of 1 kHz. The resulting *J*–*E* characteristics for all compositions exhibit a butterfly-shaped response, which is indicative of nonlinear conduction behavior typically observed in dielectric and ferroelectric materials.

As shown in Fig. 8(d)–(f), the *J*–*E* loops begin from the zero voltage, sweep through positive maximum, again reach zero, and sweep through the negative maximum before returning, forming a symmetric but hysteretic loop. This behavior suggests reversible field-dependent leakage processes, which may include trap-controlled charge injection, interfacial polarization, or space-charge-limited conduction.^60^ Among the compositions, LNS2 (Fig. 8(d)) exhibits the highest leakage current density, reaching 0.45 mA cm^−2^ at 11.60 kV cm^−1^ under the maximum applied voltage of 1500 V. This enhanced leakage can be attributed to the larger fraction of SRM in the composite, which may facilitate increased charge mobility and interfacial conduction paths due to magnetic phase boundaries or oxygen vacancies. The sharper peaks in the *J*–*E* curve near the coercive field further suggest the involvement of field-assisted domain switching and charge transport mechanisms. This suggests that the leakage mechanism with a high electric field is caused by thermally excited electrons from the metal electrode entering the conduction band of the ferroelectric phase.^61^

In contrast, the current density decreases significantly in samples with higher LNFO content. LNS3 (Fig. 8(e)) shows a reduced leakage current of 0.09 mA cm^−2^ at 11.60 kV cm^−1^, while LNS4 (Fig. 8(f)) exhibits a minimal current density of 0.03 mA cm^−2^ at 11.32 kV cm^−1^ under the same voltage conditions. The lower leakage in these compositions may be attributed to the semiconducting nature of the perovskite phase, which suppresses current flow *via* increased resistivity and reduced carrier injection at the electrode–material interface.^62^

The progressive reduction in leakage current density from LNS2 to LNS4 indicates improved dielectric insulation with increasing perovskite phase content, which is beneficial for applications requiring high breakdown strength and minimal power dissipation.^63^ Moreover, the butterfly shape and hysteresis in the *J*–*E* loops across all samples confirm the presence of polarization-related conduction phenomena and interfacial effects.^64^ As a result, the leakage current behavior supports the dielectric and ferroelectric performance trends observed in the *P*–*E* and *J*–*E* studies. To elucidate the conduction mechanism, the current density–electric field (*J*–*E*) behavior of the representative LNS2 composite was analyzed, as shown in Fig. 8(d)–(f). The *J*–*E* curve exhibits two regions: a linear regime corresponding to ohmic conduction, and a nonlinear regime, a quadratic region when the voltage is increased at higher fields. This transition signifies that the leakage process is primarily governed by space-charge-limited conduction (SCLC), in which trapped carriers within grain boundaries and interfacial defects dominate charge transport.^65^ The gradual increase in current density with voltage is consistent with trap-controlled SCLC, explaining the partial non-saturation observed in the *P*–*E* hysteresis loops. While LNS2 shows higher polarization responses, it also exhibits increased leakage, highlighting a trade-off between electromechanical performance and electrical insulation in these composites.

### 3.8. Magnetoelectric properties

#### 3.8.1. Magnetoelectric (ME) voltage response

The magnetoelectric (ME) properties of the (1 − *x*)SRM–(*x*)LNFO composites were evaluated by measuring the generated voltage response (mV) as a function of applied DC magnetic field (0–5000 Oe), under an AC magnetic field of 10 gauss at a frequency of 1 kHz. The measurements were performed under AC bias DC configuration, which is widely used to probe the direct ME effect in composite materials.

**Fig. 9:**
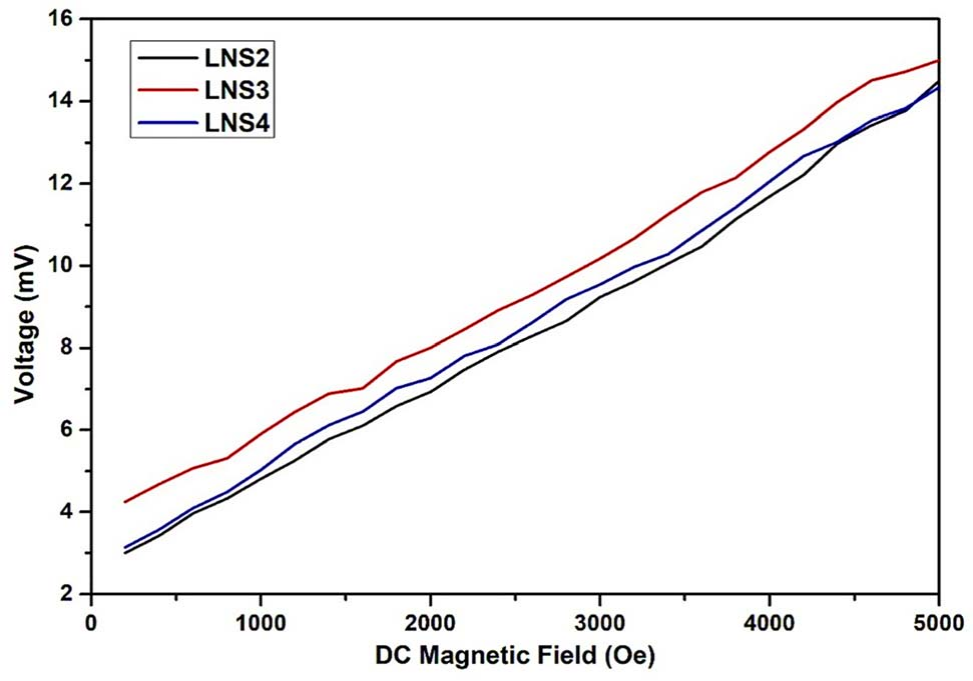
Voltage (mV) *vs.* DC magnetic field (Oe) plot for ME response.

Fig. 9 shows the voltage response of the samples (LNS2, LNS3, and LNS4) as a function of DC magnetic field. All samples exhibit a linear increase in ME voltage with the applied DC magnetic field, indicating efficient magneto-electrical coupling across the ferrite–perovskite interface. The absence of saturation or nonlinear behavior within the tested field range suggests that the ME response is governed primarily by the linear piezomagnetic and piezoelectric interactions within the composite structure.

Among the three compositions, LNS3 shows the highest ME voltage output, achieving a peak value of 15.06 mV at 5000 Oe. This enhanced ME response is attributed to the optimized phase connectivity and synergistic interplay between the magnetostrictive SRM and the piezoelectric LNFO phases. The balanced volume ratio at *x* = 0.50 likely facilitates effective mechanical strain transfer across the interface, maximizing the product tensor effect. In comparison, LNS2 and LNS4 exhibit slightly lower ME responses, recording maximum voltages of 14.52 mV and 14.26 mV at 5000 Oe, respectively. The relatively reduced ME voltage in LNS2 may result from insufficient piezoelectric contribution due to the dominance of the ferrite phase, while in LNS4, the higher content of the perovskite phase may lead to reduced magnetostrictive response. Thus, both extremities of the composition series display suboptimal phase coupling, while the intermediate composition (LNS3) exhibits the most efficient ME transduction.

The linear trend observed in all samples confirms that the ME voltage generation is field-dependent and continuous, making these composites suitable for applications in magnetic field sensing and tunable multifunctional devices.^1,5^ The results suggest that careful tuning of the magnetic-to-electric phase ratio is critical in achieving optimal ME performance in such composite systems.

#### 3.8.2. Magnetoelectric (ME) coupling behaviour

The direct magnetoelectric (ME) coupling behavior of the (1 − *x*)SRM–(*x*)LNFO composites was evaluated by measuring the ME voltage response under a low AC magnetic field (10 gauss, 1 kHz) superimposed on a varying DC magnetic field (0–5000 Oe), using an AC bias DC configuration.

**Fig. 10:**
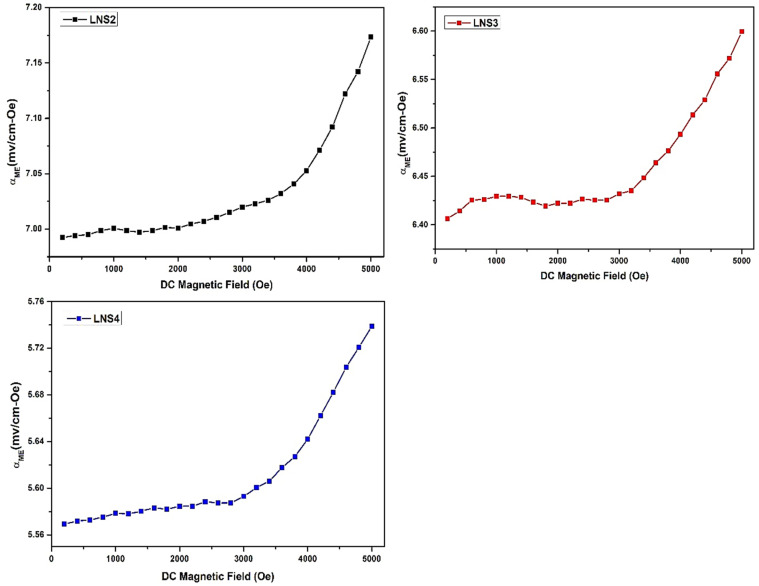
Magnetoelectric coefficient (*α*_ME_) *versus* DC magnetic field for LNS2, LNS3, and LNS4.

The magnetoelectric voltage coefficient (*α*_ME_) was calculated using the relation (2):^66^2
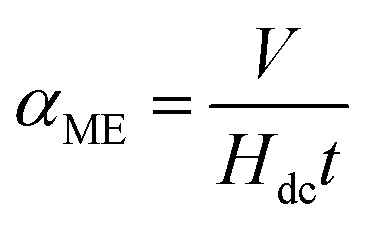
where *V* is the induced voltage across the sample, *t* is the sample thickness, and *H*_dc_ is the applied DC magnetic field. The *α*_ME_*vs. H*_dc_ plots for all three samples (LNS2, LNS3, and LNS4) are shown in Fig. 10.

All samples display a gradual, nonlinear increase in *α*_ME_ with increasing DC magnetic field, indicative of strain-mediated ME coupling driven by magnetostriction from the SRM phase and piezoelectricity from the LNFO phase. The absence of a sharp resonance-like peak and the continuous rise suggest that the ME effect is quasi-linear within the tested range, likely dominated by low-field magnetostriction and efficient interfacial strain transfer.^67,68^ Among the compositions, LNS2 exhibits the highest ME coefficient, reaching 7.17 mV cm^−1^ Oe^−1^ at 5000 Oe. The superior performance of LNS2 may be attributed to the higher concentration of the magnetostrictive SRM phase, which enhances magnetic sensitivity and mechanical deformation under applied magnetic fields. The relatively strong piezoelectric response also contributes to a more efficient ME voltage generation.^69^ LNS3, with a more balanced composition (*x* = 0.50), achieved an *α*_ME_ of 6.60 mV cm^−1^ Oe^−1^, while LNS4 exhibited the lowest value of 5.74 mV cm^−1^ Oe^−1^ at 5000 Oe. The decline in ME coefficient from LNS2 to LNS4 can be attributed to the decreasing content of the magnetic phase, which reduces the magnetostrictive strain available for ME coupling. Moreover, the increased perovskite content in LNS4 may introduce structural disorder or dampen interfacial strain transfer, thus limiting the ME response. As previously reported, the small particle size has been found to increase the mechanical connection between a composite system's two constituent phases,^70–73^ thereby improving its magnetoelectric properties.^74,75^ Moreover, the high electrical resistivity helps suppress undesirable leakage currents, which can otherwise hinder the generation of magnetoelectric (ME) voltage in the material.^76^ These results confirm that the ME performance of these composites is highly composition-dependent. To assess the effectiveness of the magnetoelectric (ME) coupling in the synthesized (1 − *x*)SrFe_12_O_19_–(*x*)La_0.5_Nd_0.5_FeO_3_ composites, a comparison with previously reported ferrite–perovskite systems was carried out, as summarized in Table 3.

**Table 3:** Comparison of magnetoelectric coupling coefficients (*α*_ME_) reported for different composite systems

Sr. no.	Composite system	ME coefficient (*α*_ME_) (mV cm^−1^ Oe^−1^)	Ref.
1	0.5(BiFeO_3_)–0.5(NiFe_2_O_4_)	6.95	77
2	Co_0.8_Ni_0.2_Fe_2_O_4_/K_0.25_Na_0.75_NbO_3_/Co_0.8_Ni_0.2_Fe_2_O_4_	3.06	78
3	Ba_0.95_Sr_0.05_TiO_3_–Ni_0.8_Co_0.2_Fe_2_O_4_	0.44	79
4	((Na_0.5_Bi_0.5_TiO_3_)–BaTiO_3_) + (Co_0.6_Zn_0.4_)(Fe_1.7_Mn_0.3_)O_4_	8.00	80
5	(0.7) Ba_0.95_Ca_0.05_Ti_0.89_Sn_0.11_O_3_–(0.3) CoFe_2_O_4_	0.10	81
6	(0.75)BaTiO_3_–(0.25)Co_0.7_Fe_2.3_O_4_	7.70	82
7	(0.95) BaFe_12_O_19_–(0.05) BiFeO_3_	2.47	83
8	(0.75)SrFe_12_O_19_–(0.25)La_0.5_Nd_0.5_FeO_3_	7.17	Present work

The optimized composition LNS2 exhibited an *α*_ME_ = 7.17 mV cm^−1^ Oe^−1^, which is significantly higher than that of reported literature as mentioned in Table 3. This enhancement confirms the role of strong magnetostrictive–piezoelectric interfacial coupling between the SrFe_12_O_19_ and La_0.5_Nd_0.5_FeO_3_ phases. The La^3+^/Nd^3+^ dual substitution in the perovskite lattice promotes localized strain and charge polarization, leading to improved magnetoelectric transduction. Hence, the present SRM–LNFO composite system demonstrates one of the highest room-temperature ME coefficients among sol–gel–derived hexaferrite–perovskite composites. Therefore, LNS2 demonstrates the strongest ME coupling, making it a promising candidate for magnetic field sensing, ME transduction, and multifunctional device applications. The observed *α*_ME_ values highlight the importance of optimizing phase connectivity and interface quality for maximizing ME output.

## 4. Conclusion

In this work, a novel series of (1 − *x*)SrFe_12_O_19_–(*x*)La_0.5_Nd_0.5_FeO_3_ (SRM–LNFO, *x* = 0.00–1.00) magnetoelectric composites was successfully synthesized *via* a citrate-assisted sol–gel method to investigate the intrinsic correlation between structural evolution and multifunctional performance. XRD and Raman analyses confirmed the coexistence and gradual phase transition between M-type hexaferrite and orthorhombic perovskite structures, while FESEM micrographs revealed composition-dependent grain refinement and enhanced densification. Mössbauer spectroscopy and XPS analyses further verified the mixed Fe^2+^/Fe^3+^ valence states and strong interfacial electronic coupling, both of which significantly influence the ferroic responses.

Magnetic studies exhibited composition-tunable coercivity and saturation magnetization, driven by interfacial strain and magnetic anisotropy modulation. Ferroelectric and leakage current analyses demonstrated that LNS2 (*x* = 0.25) and LNS3 (*x* = 0.50) achieved the most balanced electrical behavior, characterized by enhanced polarization (*P*_r_ = 1.21 µC cm^−2^), reduced leakage current density (0.45 mA cm^−2^), and superior magnetoelectric voltage coefficients (*α*_ME_ = 7.17 mV cm^−1^ Oe^−1^). The improved coupling originates from optimized phase connectivity and efficient strain-mediated charge transfer across the ferrite–perovskite interfaces. Overall, this study establishes a clear composition–microstructure–property correlation, confirming that controlled phase integration can effectively tailor multifunctional responses. The developed SRM–LNFO composites exhibit magnetoelectric coupling values underscoring their strong interfacial synergy and promising potential for magnetoelectric sensors, transducers, and electromagnetic interference (EMI) shielding devices.

## Conflicts of interest

There are no conflicts to declare.

## Supplementary Material

RA-015-D5RA07623D-s001

## Data Availability

Data will be made available upon request.
