# Supplementary material for: Compositional tuning of phase interaction and strong magnetoelectric response in SRM–LNFO ceramic composites
Source: RSC Adv. 2025 Dec 4;15(56):48006–24. doi: 10.1039/d5ra07623d (PMC12679617; doi:10.1039/d5ra07623d)
Supplement: RA-015-D5RA07623D-s001 [file RA-015-D5RA07623D-s001.pdf]

Supplementary Information (SI) for RSC Advances.
This journal is © The Royal Society of Chemistry 2025

Compositional Tuning of Phase Interaction and Strong Magnetoelectric Response in SRM-LNFO Ceramic Composites

Pramod D. Mhase [a], Varsha C. Pujari [a], Sagar E. Shirsath [b,*], Sher Singh Meena [c], Abdul Ahad [d], Santosh S. Jadhav[e], Sunil M. Patange [a,*]

[a] Department of Physics, Shri Krishna Mahavidyalaya, Gunjoti 413 606, Dharashiv, Maharashtra, India.
[b] School of Materials Science and Engineering, The University of New South Wales, Sydney, NSW 2052, Australia.
[c] Solid State Physics Division, Bhabha Atomic Research Centre, Mumbai, 400 085, Maharashtra, India
[d] Department of Pharmaceutics, College of Pharmacy, King Saud University, Riyadh 11451, Saudi Arabia
*[e] D. S. M's Arts, Commerce & Science College, Jintur, Parbhani, Maharashtra, India**

Supplementary Data

To provide a clearer understanding of the functional behavior of the intermediate compositions, the ferroelectric (P–E), leakage (J–E), and magnetoelectric (ME) data for LNS2, LNS3, and LNS4 composites are summarized in **Tables S1–S3**. These results complement the main manuscript and highlight the composition-dependent evolution of electrical and magnetoelectric properties.

The P–E hysteresis parameters (**Table S1**) show that the remanent polarization (P_r) and maximum polarization (P_{max}) values are highest for the LNS2 sample, indicating enhanced ferroelectric activity and effective dipole alignment at this composition. The observed decrease in P_r and P_{max} for LNS3 and LNS4 suggests increased electrical leakage and a reduction in long-range ferroelectric ordering as the $La_{0.5}Nd_{0.5}FeO_3$ content increases.

Table S1: Polarization–electric field (P–E) hysteresis loops of LNS2, LNS3, and LNS4 composites measured at 1 kHz and room temperature.

Sr. No.	Sample Code	P_r μC/cm^2	E_c kV/cm	P_{max} μC/cm^2
1.	LNS2	1.21	11.70	1.23
2.	LNS3	0.38	11.69	0.41
3.	LNS4	0.08	11.80	0.10

The leakage current data (**Table S2**) further confirm this trend. LNS2 exhibits a relatively higher leakage current density, likely associated with the increased charge carrier mobility near the optimized interfacial region, while LNS3 and especially LNS4 display lower J_{max} values corresponding to improved resistivity and decreased defect-assisted conduction. These differences correlate well with the P–E behavior.

Table S2: Leakage current density (J) and applied electric field (E) for LNS2, LNS3, and LNS4 composites.

Sr. No.	Sample Code	J_{max} (A/cm^2)	E_c kV/cm
1.	LNS2	0.45	11.60
2.	LNS3	0.09	11.60
3.	LNS4	0.03	11.32

The magnetoelectric voltage coefficients (**Table S3**) demonstrate the composition-dependent coupling efficiency. The maximum α_{ME} of 7.17 mV/cm.Oe for LNS2 confirms the strongest strain-mediated magnetoelectric interaction at this phase ratio, while a gradual decrease for LNS3 (6.60 mV/cm.Oe) and LNS4 (5.74 mV/cm.Oe) reflects the diminishing magnetostrictive-piezoelectric interfacial synergy at higher perovskite content.

Table S3: ME voltage coefficient (α_{ME}) at DC bias field (H_{DC}) of 5000 Oe for LNS2, LNS3, and LNS4 composites.

Sr. No.	Sample Code	ME Coefficient (α_{ME}) (mV/cm·Oe)
1.	LNS2	7.17
2.	LNS3	6.60
3.	LNS4	5.74

Overall, the supplementary results for LNS2–LNS4 confirm that the intermediate compositions exhibit optimal multifunctional behavior, arising from a balanced contribution of magnetostrictive and piezoelectric phases. The enhanced ferroelectric polarization, moderate leakage current, and highest magnetoelectric voltage coefficient observed for LNS2

demonstrate the strongest interfacial strain coupling among all samples. These findings are consistent with the trends discussed in the main manuscript and further validate the composition-dependent evolution of magnetoelectric performance across the LNS1–LNS5 series.
